# *Chaetomium atrobrunneum* causing human eumycetoma: The first report

**DOI:** 10.1371/journal.pntd.0007276

**Published:** 2019-05-30

**Authors:** Najwa A. Mhmoud, Antonella Santona, Maura Fiamma, Emmanuel Edwar Siddig, Massimo Deligios, Sahar Mubarak Bakhiet, Salvatore Rubino, Ahmed Hassan Fahal

**Affiliations:** 1 Mycetoma Research Centre, University of Khartoum, Khartoum, Sudan; 2 Faculty of Medical Laboratory Sciences, University of Khartoum, Khartoum, Sudan; 3 Department of Biomedical Sciences, University of Sassari, Sassari, Italy; 4 Institute for Endemic Diseases, University of Khartoum, Khartoum, Sudan; Rutgers University, UNITED STATES

## Abstract

In this communication, a case of black grain eumycetoma produced by the fungus *C*. *atrobrunneum* is reported. The patient was initially misdiagnosed with *M*. *mycetomatis* eumycetoma based on the grains’ morphological and cytological features. However, further aerobic culture of the black grains generated a melanised fungus identified as *C*. *atrobrunneum* by conventional morphological methods and by internal transcribed spacer 2 (ITS2) ribosomal RNA gene sequencing. This is the first-ever report of *C*. *atrobrunneum* as a eumycetoma-causative organism of black grain eumycetoma. It is essential that the causative organism is identified to the species level, as this is important for proper patient management and to predict treatment outcome and prognosis.

## Overview

Mycetoma is a chronic, progressive, granulomatous, subcutaneous inflammatory disease. It is caused by certain fungi and bacteria, and thus, it is classified as a eumycetoma and an actinomycetoma, respectively. More than 50 microorganisms were reported as mycetoma-causative organisms. *Madurella mycetomatis* is the most frequently reported eumycetoma-causative organism, with the highest worldwide disease burden. It generally affects children and young adults of low socioeconomic status, causing devastating deformities, disability, and high morbidity in late disease, and it has many severe effects on patients and communities. In this communication, we report on a 32-year-old patient from the White Nile state, Central Sudan. He presented with a painless subcutaneous swelling in the left foot of 7 years’ duration. The clinical diagnosis was *M*. *mycetomatis* eumycetoma, for which he underwent wide surgical excision. The black grains’ aerobic culture generated a melanised fungus identified as *Chaetomium atrobrunneum* by conventional morphological methods and by internal transcribed spacer 2 (ITS2) ribosomal RNA gene sequencing. The patient was started on empiric itraconazole, and fungal susceptibility was later confirmed by Sensititre YeastOne test (minimum inhibitory concentration [MIC] ≤ 0.06). No evidence of recurrence was observed after 1 year of treatment. Our study adds *C*. *atrobrunneum* from the Sordariales order and Chaetomiaceae family to the list of melanised fungi causing human black grain eumycetoma. Both phenotypic and genetic methods were fundamental to elucidate the eumycetoma associated with this unusual mould and to determine the appropriate therapy.

## Introduction

Mycetoma is a chronic granulomatous inflammatory disease that is characterised by local swelling and draining sinuses that drain grains of different colour and size depending on the causative agent; the disease is caused either by bacteria or fungus, with the latter being the most common causative agent of mycetoma reported in Sudan [1 – 3]. The two major factors for successful management of mycetoma patients are better identification of the causative agents and better prevention and treatment of infection [2, 4]. The identification of the causative agent is of value for proper treatment and identification of the drug of choice for patient treatment [5].

*Chaetomium* species are scattered worldwide, in animal dung, straw, paper, bird feathers, seeds, plant debris, and soil [6]. The genus, *Chaetomium*, encompasses more than 100 species, most of which grow best in a temperature ranging from 25–37°C [7, 8]. This fungus is able to affect healthy and immunocompromised people; the most common pathogenic species was *Chaetomium globosum* [8 – 10]. Clinical features of infection have included those in association with onychomycosis [8, 9], keratitis [11, 12], sinusitis [13], lung empyema [14], pneumonia, and fatal disseminated cerebral mycosis [15]. However, *C*. *atrobrunneum* has not been previously reported to be associated with mycetoma infection.

### Case report

The patient is a 32-year-old farmer from the White Nile, Central Sudan, who presented in 2010 to the Mycetoma Research Centre (MRC), Khartoum, Sudan, with a painless left foot swelling of 4 years’ duration. His condition started with a small, painless subcutaneous swelling on the heel of the left foot that gradually increased in size. In 2008, it was diagnosed as an abscess, for which he underwent surgical drainage under local anaesthesia at a district general hospital twice.

The patient had no medical comorbidities and had no family history of similar conditions. He had an ultrasound examination of the swelling, which showed a surgical scar and a single cavity that contained fluid collection and echogenic aggregated grains suggestive of residual mycetoma. He underwent fine needle aspiration for cytology, which showed black grains and inflammatory infiltrates in line with *M*. *mycetomatis* with type I and II tissue reactions. He was started on 400 mg of ketoconazole twice per day (BID), and he was on regular follow-up in the MRC for 18 months; he then dropped the follow-up and treatment.

In 2017, he was seen at El Andalous Health Centre, the White Nile, with the same left foot lesion, which had increased in size. On examination, he looked well and not pale. He was haemodynamically stable. The systemic examinations were unremarkable. Local examination showed a firm subcutaneous mass on the left heal, which was 4 × 4 cm, firm in consistency, and attached to the skin and had deep structures with multiple sinuses and discharge of black grains.

His liver function test showed serum bilirubin of 0.3 mg/dL, total protein of 8 g/d/L, serum albumin of 5 g/dL, alkaline phosphatase of 98 U/L, aspartate aminotransferase (AST) of 15 U/L, and alanine aminotransferase (ALT) of 20 U/L. His renal function test showed normal blood urea of 21 mg/dL and serum creatinine of 0.51 mg/dL. His complete blood count examination showed leucocytosis, with a total white blood cell count of 12.0 × 10^3^, haemoglobin count of 12.1 g/dL, and platelet count of 397 × 10^3^. Lesion ultrasound examination findings were in line with eumycetoma. He underwent wide local excision of the mass under spinal anaesthesia with uneventful postoperative recovery. He was started on 200 mg BID of itraconazole and 5 mg daily of folic acid and received daily wound dressing.

**Fig 1:**
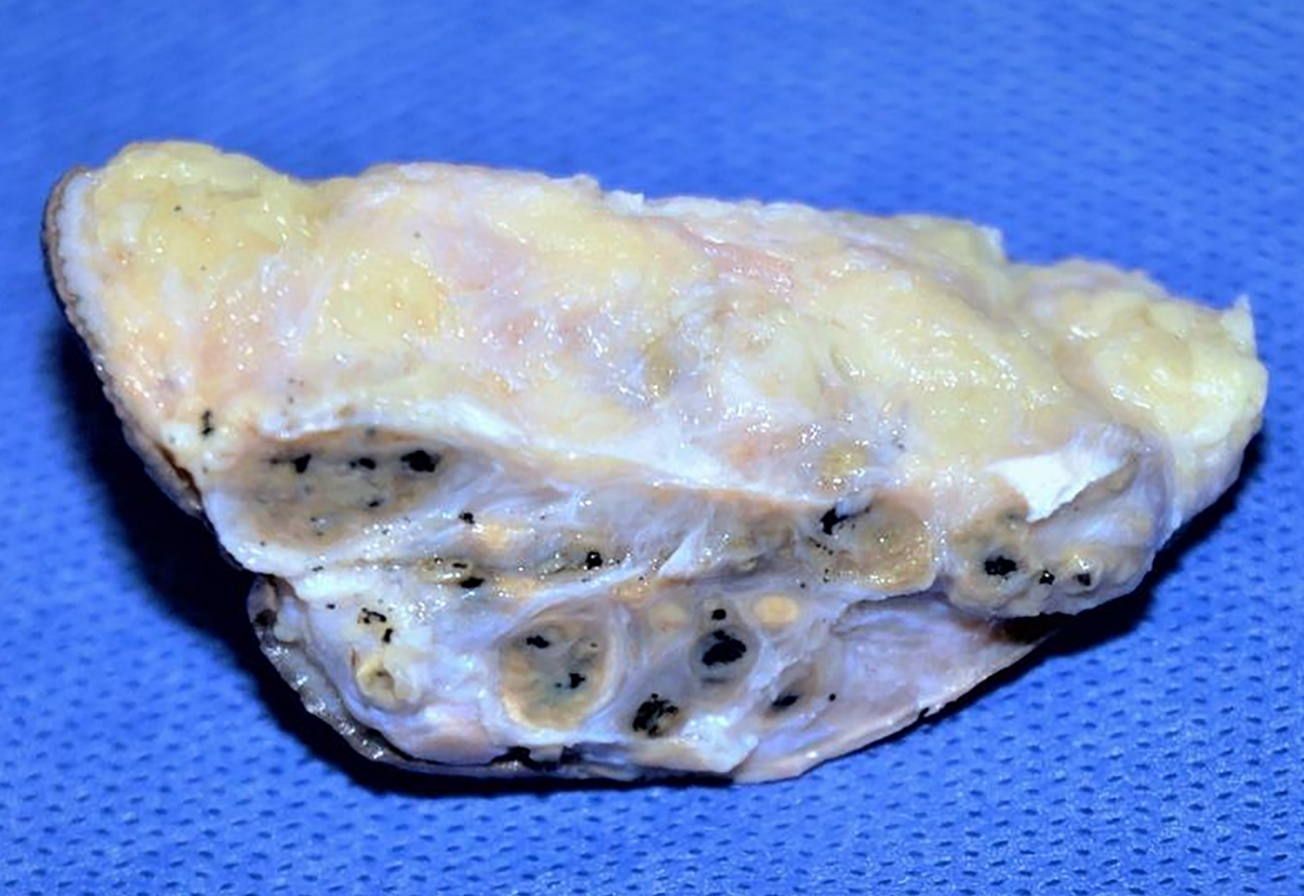
Microphotograph showing the grossing appearance of the lesion that showed multiple black grains.

**Fig 2:**
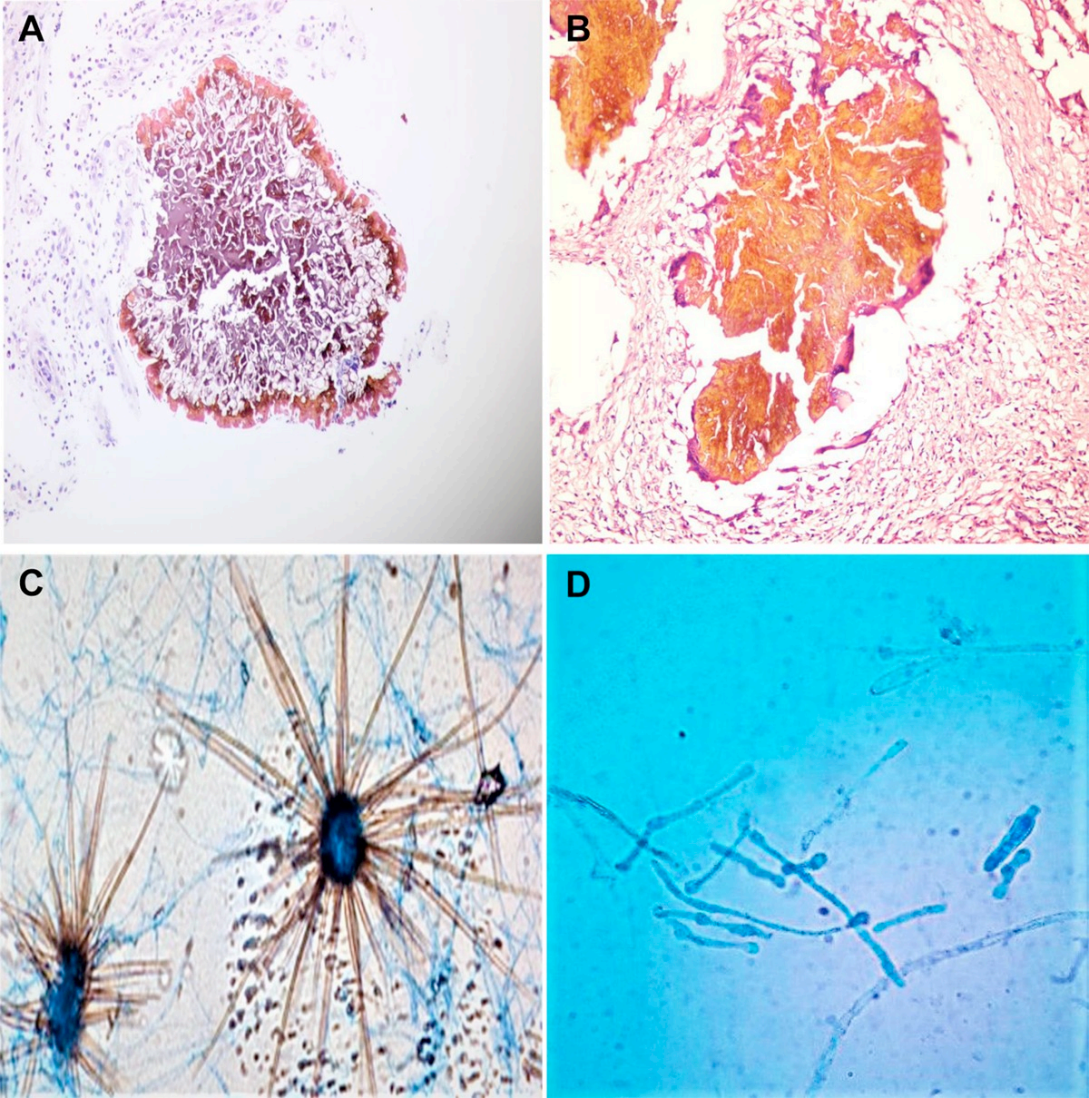
Microphotograph showing multiple black grains surrounded by granulation tissue with marked histiocytic and mixed inflammatory cellular infiltrates (HE 10×). (A) Grains of *Chaetomium* spp. showed abundant extracellular matrix, which was yellow to brown in colour, and the fungus hyphae are located at the periphery of the matrix with short filamentous structure. (B) The filamentous pattern of *M*. *mycetomatis* grains consists of brown septate and branched hyphae at the centre and periphery with long filament. (C, D) Microphotograph of LPCB mount showing ascoma and ascospore cells resembling the typical *Chaetomium* spp. cells (C) and conidia of *M*. *mycetomatis* (D). HE, haematoxylin–eosin; LPCB, lactophenol cotton blue.

The surgical biopsy and grains were persevered partly in normal saline for grain culture and partly in 10% formal saline for histopathological examination. A paraffin-processed tissue block, which measured 6.5 × 3.8 × 1.5 cm, was prepared from the surgical biopsy (Fig 1). The tissue block was cut using a rotary microtome, and subsequently, 3–5 μm sections were obtained. The sections were stained with haematoxylin–eosin (HE) stain. Microscopical examination showed multiple black grains surrounded by granulation tissue. There were marked histiocytic and mixed inflammatory cellular infiltrates, in line with *M*. *mycetomatis* eumycetoma; however, a few differences were observed compared with *M*. *mycetomatis* eumycetoma (Table 1) [16]. There was an abundant extracellular matrix, which was yellow to brown in colour, and the fungus hyphae were seen at the periphery of the matrix as short filamentous structures, leaving an empty, cracking centre that contains only cement matrix infiltrated by neutrophils (Fig 2A and 2B).

**Table 1:** The different characteristic features of *C*. *atrobrunneum* and *M*. *mycetomatis*.

Eumycetoma features	*C*. *atrobrunneum*	*M*. *mycetomatis* [16]
**Host immune reaction**	Chronic granuloma	Chronic granuloma
**Cement matrix**	Abundant with yellow to brown colour	Abundant with brown colour
**Fungus hyphae**	Periphery of the matrix with short filamentous structure	At the centre and periphery with long filament

The black grains were washed three times in saline solution and then cultivated on nonselective and selective media that included blood agar (BA), potato agar (PA), Sabouraud dextrose agar (SDA) with and without chloramphenicol (0.05 g/L), and gentamicin (0.1 g/L) at 37°C. Fungal growth appeared after 3 days in BA and 5 days on PA and SA. Microscopic examination of lactophenol cotton blue (LPCB) mount showed ascoma and ascospore cells resembling the typical *Chaetomium* spp. cells from the Chaetomiaceae family (Fig 2C and 2D).

DNA was extracted from the isolate cultured in Sabouraud liquid media (Oxoid) using YeaStar Genomic DNA Kit (Zymo Research, Tustin, California; United States); it was then amplified by PCR using specific ITS2 rRNA primers for fungi [17]. The specific amplicon was purified using DNA clean and concentrator (TM-5-Zymo Research, California, US) and were Sanger sequenced (BMR Genomics, Padova). Sequences were analysed using Geneious 11 software (http://www.geneious.com/), and the species was identified by BLAST database sequences comparison. A 570 bp sequence was obtained from the purified ITS2 amplicon, and it showed 99.6% identity with *C*. *atrobrunneum* (KX146507).

The patient was started empirically on 400 mg per day of itraconazole in two divided doses and 5 mg of folic acid once daily. Antifungal susceptibility testing was performed using the YeastOne (Sensititre; Thermo Scientific, Cleveland, Ohio, USA) system test as previously described for *Aspergillus* spp. [18]. For the in vitro susceptibility test, we used a commercial colorimetric microdilution assay; the isolate was first subcultured in SDA and incubated for 7 days at 35°C to obtain adequate sporulation. After that, we collected the conidia using a sterile cotton swab, suspended it in sterile normal saline with 10% Tween, and obtained the correct turbidity of 0.5 McFarland standard. Then, we added 100 μL of the suspension to 11 mL of YeastOne inoculum broth and incubated the plate at 35°C for 48 hours. After 24 hours, we observed the positive control and the control in the SDA. For *Chaetomium* spp., we read the MIC as the lowest concentration with a blue colour. The interpretations of the MIC are based on the European Committee on Antimicrobial Susceptibility Testing (EUCAST) breakpoint.

*C*. *atrobrunneum* proved to be susceptible (S) to itraconazole, with MIC of S ≤ 0.06, (Table 2). The patient is on regular follow-up at the MRC, and he had no evidence of recurrence after 1 year of treatment.

**Table 2:** MIC of antifungal agents against *C*. *atrobrunneum* MRC9 isolate by Sensititre YeastOne test.

Antifungal agent	MIC	
Itraconazole		S ≤ 0.06
Posaconazol		S ≤ 0.06
Caspofungin		S ≤ 0.06
Mycafungin		S ≤ 0.06
Anidulafungin		S ≤ 0.05
Amphotericin		R > 4
5-Fluorocitosin		R = 8
Fluconazole		R = 256

Abbreviations: MIC, minimum inhibitory concentration; R, resistance; S, susceptible.

The evolutionary history of the *C*. *atrobrunneum* strain isolated here was inferred using the neighbor-joining method [19], including ITS nucleotide sequences of *Chaetomium* and *Madurella* species (*n* = 66) of both clinical and environmental origins. The optimal tree had a branch length sum of 1.09302367. The percentage of replicate trees in which the associated taxa clustered together in the bootstrap test (1,000 replicates) is shown next to the branches [20]. The tree is drawn to scale, with branch lengths in the same units as those of the evolutionary distances used to infer the phylogenetic tree. The evolutionary distances were computed using the maximum composite likelihood method [21] and are in units of the number of base substitutions per site. All ambiguous positions were removed for each sequence pair (pairwise deletion option). There were a total of 666 positions in the final dataset. Evolutionary analyses were conducted in Molecular Evolutionary Genetics Analysis (MEGA) X [22].

**Fig 3:**
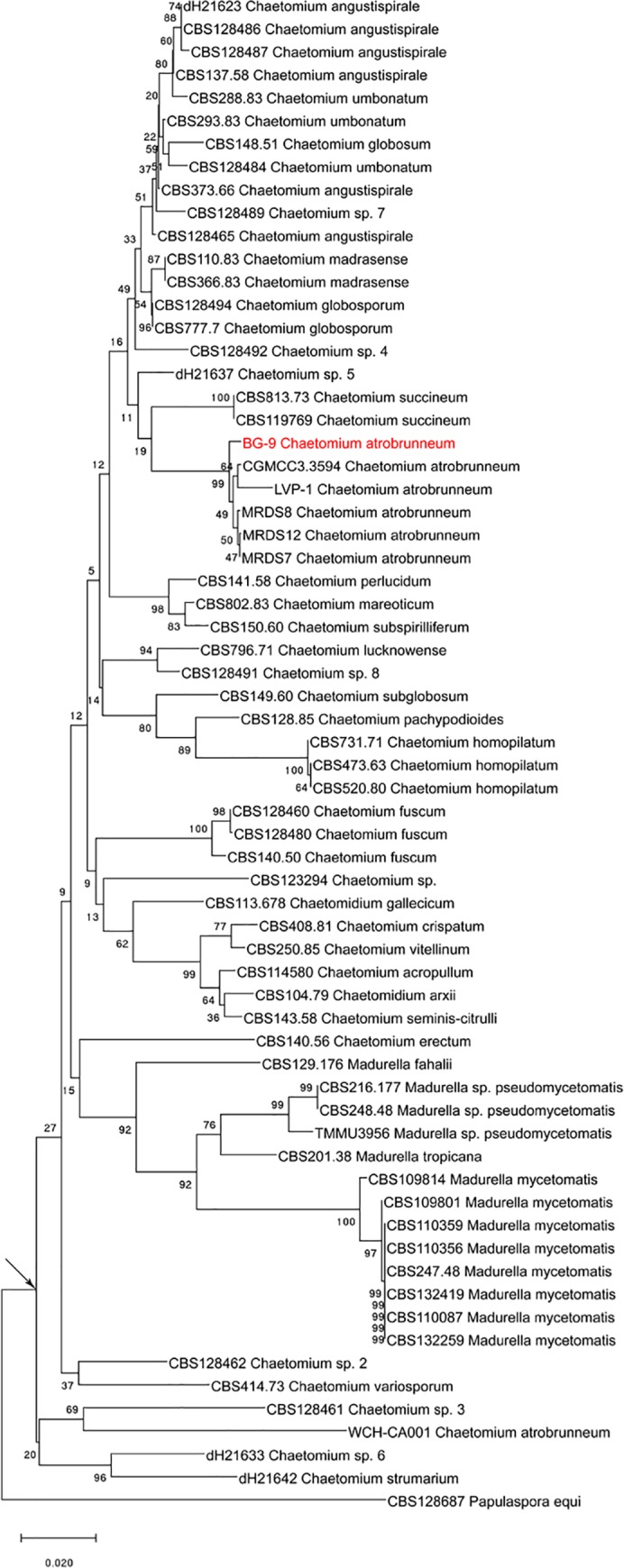
Neighbor-joining phylogenetic tree of ITS sequences of *C*. *atrobrunneum* BG9 strain (in red) and *Chaetomium* and *Madurella* strains of both clinical and environmental origin, downloaded from NCBI Database. A *Papulaspora equi* strain (CBS 128687) was used to root the tree. The black arrow indicates the *Chaetomium* clade. ITS, internal transcribed spacer; NCBI, National Center for Biotechnology Information.

The phylogenetic tree showed *Madurella* inside the *Chaetomium* clade (Fig 3), with *C*. *atrobrunneum* BG9 closely related to the other *C*. *atrobrunneum* strains isolated from plants (MRDS7, MRDS8, and MRDS12) and from human (LVP-1), and *C*. *succineum* strains also isolated from plants. Only one *C*. *atrobrunneum* strain (WCH-CA001 ITS, human origin) clustered separately from the other *C*. *atrobrunneum*, with few *Chaetomium* spp.

## Discussion

Mycetoma is a badly neglected medical and health disease endemic in many tropical and subtropical countries around the world [23 – 25]. Certain environment factors such as rainfall, humidity, and temperature influence the geographical disease distribution [26 – 28]. One-third of all the reported mycetomas worldwide were from Sudan, where eumycetoma accounts for 70% of these cases and *M*. *mycetomatis* is the most frequently reported [29 – 31]. Mycetoma is believed to occur as a result of traumatic implantation of the causative organism into the subcutaneous tissue through minor trauma [26]. It then spreads to involve the skin, deep tissues, and bone, leading to massive destruction, deformities, and disabilities [32, 33]. If untreated, it can have a major impact on the affected patients, communities, and health system in endemic counties [34, 35].

In this communication, we reported and described the first case of black grain eumycetoma caused by *C*. *atrobruneumm*, a member of the Chaetomiaceae family and Sordariales order, which was initially clinically diagnosed as *M*. *mycetomatis* eumycetoma, the most frequent cause of eumycetoma worldwide and, in particular, in Sudan [36].

Fungi species causing eumycetoma belong to seven different orders (Table 3), with Sordariales, Pleiorales, and Chaetothyriales able to produce black grains (Table 3).

**Table 3:** Eumycetoma-causing agents.

Eumycetoma species	Order	Family	Grain	Ref
*Exophiala jeanselmei*	Chaetothyriales	Herpotrichiellaceae	Black	[47]
*Phaeoacremonium krajdenii*	Diaporthales	Togniniaceae	White	[48]
*Phaeoacremonium parasiticum*	Diaporthales	Togniniaceae	White	[49]
*Aspergillus flavus*	Eurotiales	Aspergillaceae	White	[50]
*Aspergillus hollandicus*	Eurotiales	Aspergillaceae	White	[51]
*Aspergillus nidulans*	Eurotiales	Aspergillaceae	White	[52]
*Acremonium recifei*	Hypocreales	Nectriaceae	White	[53]
*Cylindrocarpon cyanescens*	Hypocreales	Nectriaceae	White	[54]
*Cylindrocarpon destructans*	Hypocreales	Nectriaceae	White	[55]
*Fusarium falciforme*	Hypocreales	Nectriaceae	White	[56]
*Fusarium solani*	Hypocreales	Nectriaceae	White	[57]
*Fusarium verticillioides*	Hypocreales	Nectriaceae	White	[56]
*Acremonium kiliense*	Hypocreales	Incertae sedis	White	
*Acremonium potronii*	Hypocreales	Incertae sedis	White	
*Phialemonium obovatum*	Hypocreales	Cephalothecaceae	White	[57]
*Scedosporium boydii*	Microascales	Microascaceae	White	[58]
*Microsporum canis*	Onygenales	Arthrodermataceae	White	
*Trichophyton* sp.	Onygenales	Arthrodermataceae	White	
*Neotestudina rosatii*	Pleosporales	Testudinaceae	White/black	[28]
*Bipolaris spicifera*	Pleosporales	Pleosporaceae	Black	
*Curvularia geniculata*	Pleosporales	Pleosporaceae	Black	[59]
*Curvularia lunata*	Pleosporales	Pleosporaceae	Black	[60]
*Medicopsis romeroi*	Pleosporales	Neohendersoniaceae	Black	[61]
*Falciformispora senegalensis*	Pleosporales	Leptosphaeriaceae	Black	[62]
*Falciformispora tompkinsii*	Pleosporales	Leptosphaeriaceae	Black	[62]
*Pseudochaetosphaeronema larense*	Pleosporales	Incertae sedis	Black	[62]
*Corynespora cassiicola*	Pleosporales	Corynesporascaceae	Black	[28]
*Nigrograna mackinnonii*	Pleosporales	Nigrogranaceae	Black	[28]
*Madurella grisea*	Pleosporales	Chaetomiaceae	Black	[28]
*Madurella fahalii*	Sordariales	Chaetomiaceae	Black	[28]
*M*. *mycetomatis*	Sordariales	Chaetomiaceae	Black	[28]
*Madurella pseudomycetomatis*	Sordariales	Chaetomiaceae	Black	[28]
*Madurella tropicana*	Sordariales	Chaetomiaceae	Black	[28]
*C*. *atrobrunneum*	Sordariales	Chaetomiaceae	Black	This study

More than 100 *Chaetomium* species from the ascomycete Chaetomiaceae family were reported. Most of them can produce intricate fruiting bodies with characteristically shaped setae and ascospores, making them microscopically distinguishable from *Madurella* and other Sordariales species.

*Chaetomium* species commonly reside in soil enriched with animal dung or cellulosic materials and also in indoor environments [37]. Only a few cases of *C*. *atrobrunneum* human infections have been previously reported worldwide [38, 39]. This infection can cause minor disorders such as allergic reaction, onychomycosis, and sinusitis. In immunocompromised patients and bone marrow transplant recipients, it can cause serious and fatal infections such as empyema [40], pneumonia, and fatal disseminated cerebral disease [37].

*C*. *atrobrunneum* was rarely reported in eye infections. It was reported as a cause of keratitis in an adult male [41] and retinitis in a patient with Hodgkin lymphoma [42].

It was also reported in mixed infections, including cutaneous eyelid infection caused by *C*. *atrobrunneum* and *Clavispora lusitaniae*, [43] and in a fatal pneumonia caused by *C*. *atrobrunneum* and *Aspergillus fumigatus* [44].

The appropriate treatment for *Chaetomium* infections is unknown. Published in vitro susceptibility data for *Chaetomium* species have revealed resistance to flucytosine and fluconazole [45]. In the case reported here, *C*. *atrobrunneum* isolate was susceptible to itraconazole with a low MIC.

In the last 20 years, significant progress in fungal systematics and taxonomy has been achieved because of advancement in the next-generation sequencing technologies and bioinformatic tools [46].

In a recent phylogenetic study, the genus *Madurella*, comprising the species *M*. *mycetomatis*, *M*. *pseudomycetomatis*, *M*. *fahalii*, and *M*. *tropicana*, was found to cluster with *C*. *atrobrunneum* and other *Chaetomium* spp. within the Chaetomiaceae family. Here, we showed that *M*. *mycetomatis* and *C*. *atrobrunneum* are not only phylogenetically but also clinically related, causing human eumycetomas that are clinically indistinguishable and identifiable using both phenotypic and genetic methods.

In conclusion, we reported on the first human eumycetoma caused by *C*. *atrobrunneum*, adding another Sordariales species from Chaetomiaceae, such as *M*. *mycetomatis*, to the list of melanised fungi that cause human black grain mycetoma. This new case of eumycetoma confirmed *Chaetomium* spp.’s inclination to cause human infection, which needs to be explored.

### Ethics statement

The study was approved by the Mycetoma Research Center Institutional Review Board (IRB) (5/2018). Written, informed consent to publish history, findings, and images for educational purposes was obtained from the patient.

**Key learning points**

- *C*. *atrobrunneum* is a rare cause of eumycetoma in Sudan.
- The histopathological discrimination between *C*. *atrobrunneum* grains and *M*. *mycetomatis* is frequently difficult and can be misleading.
- Molecular identification of mycetoma causative agent to the species level is mandatory.
